# Deletion of the *App-Runx1* region in mice models human partial monosomy 21

**DOI:** 10.1242/dmm.017814

**Published:** 2015-06-01

**Authors:** Thomas Arbogast, Matthieu Raveau, Claire Chevalier, Valérie Nalesso, Doulaye Dembele, Hugues Jacobs, Olivia Wendling, Michel Roux, Arnaud Duchon, Yann Herault

**Affiliations:** ^1^ Institut de Génétique et de Biologie Moléculaire et Cellulaire, Department of Translational Medicine and Neurogenetics, 1 rue Laurent Fries, Illkirch 67404, France; ^2^ Centre National de la Recherche Scientifique, UMR7104, Illkirch 67404, France; ^3^ Institut National de la Santé et de la Recherche Médicale, U964, Illkirch 67404, France; ^4^ Université de Strasbourg, Illkirch 67404, France; ^5^ Institut Clinique de la Souris, PHENOMIN-ICS, CNRS, INSERM, Université de Strasbourg, 1 rue Laurent Fries, Illkirch 67404, France

**Keywords:** Mouse model, Aneuploidy, Learning and memory, Motor coordination

## Abstract

Partial monosomy 21 (PM21) is a rare chromosomal abnormality that is characterized by the loss of a variable segment along human chromosome 21 (Hsa21). The clinical phenotypes of this loss are heterogeneous and range from mild alterations to lethal consequences, depending on the affected region of Hsa21. The most common features include intellectual disabilities, craniofacial dysmorphology, short stature, and muscular and cardiac defects. As a complement to human genetic approaches, our team has developed new monosomic mouse models that carry deletions on Hsa21 syntenic regions in order to identify the dosage-sensitive genes that are responsible for the symptoms. We focus here on the Ms5Yah mouse model, in which a 7.7-Mb region has been deleted from the *App* to *Runx1* genes. Ms5Yah mice display high postnatal lethality, with a few surviving individuals showing growth retardation, motor coordination deficits, and spatial learning and memory impairments. Further studies confirmed a gene dosage effect in the Ms5Yah hippocampus, and pinpointed disruptions of pathways related to cell adhesion (involving *App*, *Cntnap5b*, *Lgals3bp*, *Mag*, *Mcam*, *Npnt*, *Pcdhb2*, *Pcdhb3*, *Pcdhb4*, *Pcdhb6*, *Pcdhb7*, *Pcdhb8*, *Pcdhb16* and *Vwf*). Our PM21 mouse model is the first to display morphological abnormalities and behavioural phenotypes similar to those found in affected humans, and it therefore demonstrates the major contribution that the *App-Runx1* region has in the pathophysiology of PM21.

## Abstract (precis)

**Summary:** The Del(16*App-Runx1*)5Yah mouse model displays morphological abnormalities and behavioural phenotypes similar to those found in humans with partial monosomy 21, and it therefore demonstrates the major contribution of the *App-Runx1* region to the pathophysiology of partial monosomy 21.

## INTRODUCTION

Segmental aneuploidy, which is defined as an abnormal copy number of a genomic region, is a common cause of human genetic disorders and often leads to intellectual disabilities. Human chromosome 21 (*Homo sapiens* 21, Hsa21) aneuploidies are associated with trisomy 21 or Down syndrome, which is the principal genetic cause of intellectual disabilities. Although extremely rare, different cases of partial Hsa21 monosomies (PM21) have been reported since 1964, when Lejeune described the first PM21 case for a small acrocentric chromosome (Lejeune et al., 1964). Modern techniques have confirmed that the complete monosomy of Hsa21 without any translocation to another chromosome is incompatible with life (Burgess et al., 2014; Toral-Lopez et al., 2012). Depending on their size and location on Hsa21, partial deletions are associated with a large heterogeneity of clinical phenotypes. Some affected individuals present with severe phenotypes, such as brain dysgenesis and heart defects that are not compatible with survival; others show milder phenotypes, such as slight dimorphic features or no symptoms at all. The most common features of PM21 include intellectual disability, craniofacial malformations, short stature, and muscular and cardiac defects (Chettouh et al., 1995; Lindstrand et al., 2010; Lyle et al., 2009; Roberson et al., 2011; Theodoropoulos et al., 1995; Valero et al., 1999).

The first molecular mapping of features that are associated with PM21 was performed in 1995, and compared the phenotypes and karyotypes of six individuals (Chettouh et al., 1995). The analysis pinpointed a 5.3-Mb region from *APP* to *SOD1* that is involved in intellectual disability, hypotonia and cranio-facial malformations. However, high-resolution mapping of pathogenic partial aneuploidies and unbalanced translocations involving Hsa21 do not indicate that a single region is crucial; instead, they reveal susceptible regions for the different phenotypes of PM21 (Lindstrand et al., 2010; Lyle et al., 2009; Roberson et al., 2011). The long arm of Hsa21 can roughly be divided into three regions (Lyle et al., 2009). The first region, which stretches from the centromere to approximately 31.2 Mb, covers a gene-poor region of Hsa21 (approximately 50 genes). Only large deletions are found in affected individuals that exhibit intellectual disability, muscular defects and several cranio-facial malformations. The second region, which spans from 31.2 to 36 Mb, has a high gene density (approximately 80 genes). Few individuals carrying a partial deletion have been diagnosed with severe phenotypes, which indicates that the haploinsufficiency of the entire region might not be compatible with survival. In the last region, which stretches from 36 Mb to the telomere (approximately 130 genes), deletions induce relatively mild phenotypes. Given the rarity of such individuals, it is very difficult to identify genes that are responsible for the different PM21 symptoms. Complementary to the genetic analysis, mouse models have been developed to study the correlation between phenotype and genotype. Almost all of the protein-coding genes found on the Hsa21 long arm have homologues that are carried by mouse chromosomes 16 (*Mus musculus* 16, Mmu16; 23.3 Mb, 166 genes between *Lipi* and *Zfp295*), 17 (Mmu17; 1.1 Mb, 22 genes between *Umodl1* and *Hsf2bp*) and 10 (Mmu10; 2.3 Mb, 45 genes between *Pdxk* and *Prmt2*). This synteny allows mouse models to be used to facilitate the identification of the genes responsible for the symptoms of PM21 individuals.

**TRANSLATIONAL IMPACT**

**Clinical issue**

Partial monosomy 21 (PM21) is a rare chromosomal abnormality that is characterized by the loss of a variable segment on the long arm of chromosome 21 (Hsa21q). Depending on the size and location of the deletions, PM21 is associated with a large range of heterogeneous clinical phenotypes, some of which are fatal. The most common features of PM21 include intellectual disability and craniofacial malformations, as well as muscular, skeletal and cardiac abnormalities. There is currently no treatment to alleviate the individual conditions, and the development of therapeutic strategies will require a broader understanding of the disease. To identify candidate genes, several mouse models deleted for different Hsa21q homologous regions have been established. However, these models all show relatively mild phenotypes. In this context, the value of a new animal model that recapitulates the most severe features of PM21 cannot be understated.

**Results**

In this study, the authors characterize the Ms5Yah mouse model, which is deleted for the *App-Runx1* region (homologous to the Hsa21q21.3-22.11 region). In previous work, the authors have reported the importance of this interval in cardiac defect phenotypes of a Down syndrome mouse model. Here, they report that the Ms5Yah mouse model exhibits developmental delays that affect viability, size and weight. Viability tests and histological analyses indicate that the majority of mutant neonates show impaired breathing. Haematology analysis reveals a platelet deficit, which has been reported in some individuals with PM21, and behavioural studies reveal severe impairments in motor coordination and spatial learning, as well as memory deficits. Finally, analysis of gene expression in the hippocampus, the brain region responsible for these functions, pinpoints a disruption of cell adhesion pathways.

**Implications and future directions**

Anatomical and behavioural characterization of Ms5Yah mice suggests that the *App-Runx1* region has a major impact on the most severe phenotypes of PM21. Specifically, behavioural characterization of Ms5Yah mice indicates locomotor learning deficits in addition to spatial learning and memory deficits, which can be directly related to the intellectual disabilities of individuals with PM21. The present mouse model therefore represents a novel genetic tool to identify pathways that are implicated in the pathophysiology of PM21. Moreover, future studies dedicated to the search for therapeutic agents that rescue lethality and/or learning impairment of Ms5Yah mice could provide the first treatment strategies for partial monosomy 21.

To date, several mouse models carrying deletions on Mmu17 and Mmu10 syntenic regions corresponding to the distal part of human 21q22.3 have been generated and characterized. Ms1Yah mice carrying a 0.5-Mb deletion between *Prmt2* and *Col61a* genes on Mmu10 show no behavioural phenotype but exhibit an increased inflammatory reaction after intranasal lipopolysaccharide administration (Besson et al., 2007); Ms4Yah mice deleted for the 2.2 Mb *Prmt2*-*Cstb* region on Mmu10 present no gross dimorphism or behavioural defects (Duchon et al., 2011). Del(10*Prmt2-Pdxk*)4Yey mice carrying a 2.3-Mb deletion of the entire Mmu10 syntenic region between *Prmt2* and *Pdxk*, which is directly upstream from *Cstb*, show moderate spatial memory impairment (Yu et al., 2010). Del(17*Abcg1-Rrp1b*)5Yey mice deleted for a 1.1-Mb region on Mmu17 between *Abcg1* and *Rrp1b* present deficits in associative memory (Yu et al., 2010). A recent study has shown that Del(17*Abcg1-Cbs*)2Yah mice deleted for the 0.59-Mb *Abcg1-U2af1* region display decreased social novelty interactions and alterations in hippocampic long-term potentiation (Sahún et al., 2014). Two mouse models carrying deletions in the Mmu16 syntenic region have been generated and characterized previously. Del(16*Cbr1-Fam3b*)1Rhr mice deleted for the 4.2-Mb *Cbr1-Fam3b* region syntenic to Hsa21, which is called the ‘Down syndrome critical region’ (Epstein, 1990; Korenberg et al., 1994), display no behavioural phenotype but show decreases in the volumes of the brain and hippocampus, as well as an increase in the volume of the cerebellum (Olson et al., 2007). Ms1Dja mice harbouring a 1.6-Mb deletion between *Lipi* and *Usp25*, corresponding to the Hsa21 centromeric region 21q11.2-q21.1, exhibit increased fat deposition and moderate social memory impairment (Migdalska et al., 2012).

To study the influence of the Hsa21q21.3-22.11 homologous region, the Del(*16App-Runx1*)5Yah (also named Ms5Yah) mouse model carrying a deletion of the 7.7 Mb *App-Runx1* genetic interval was engineered in our lab. In 2012, Raveau et al. demonstrated how this region influences the lethality and cardiac defects observed in the Ts65Dn Down syndrome mouse model (Raveau et al., 2012). In the present study, we characterize the Ms5Yah mouse model and explore both its impact on postnatal viability, and its consequences on behaviour and cognition. An important lethality was observed at birth and in the first days postpartum. At weaning, only 22.4% of the animals were carrying the *App-Runx1* deletion. Compared with their control littermates, Ms5Yah mice showed decreased body size, weight reduction and platelet deficits. Defects were identified in the rotarod, the notched bar test and in the Morris water maze. To understand the possible underlying mechanisms in the hippocampus, we performed a transcriptomic analysis and observed a whole-genome effect from the decrease in gene dosage associated with the *App-Runx1* region. Overall, our observations demonstrated that the Ms5Yah mouse model recapitulates the major symptoms observed in PM21 in humans.

## RESULTS

### Loss of one copy of the *App-Runx1* region impacts early postnatal viability, basic behaviour and general morphology

During the breeding of Ms5Yah mice, we experienced major difficulties in establishing the monosomic model on a pure C57BL/6N genetic background. Thus, we maintained the line on a B6C3B mixed genetic background. Despite a marked improvement over the C57BL/6N background, we noticed a low transmission of the Ms5Yah allele at weaning. Indeed, of the 389 mice generated from a wild-type and a heterozygote carrier, 87 Ms5Yah individuals were observed at weaning. The 22.4% allele frequency was far below the 50% that could be expected from a Mendelian ratio (Chi-squared test, *P*<0.001).

**Fig. 1.**
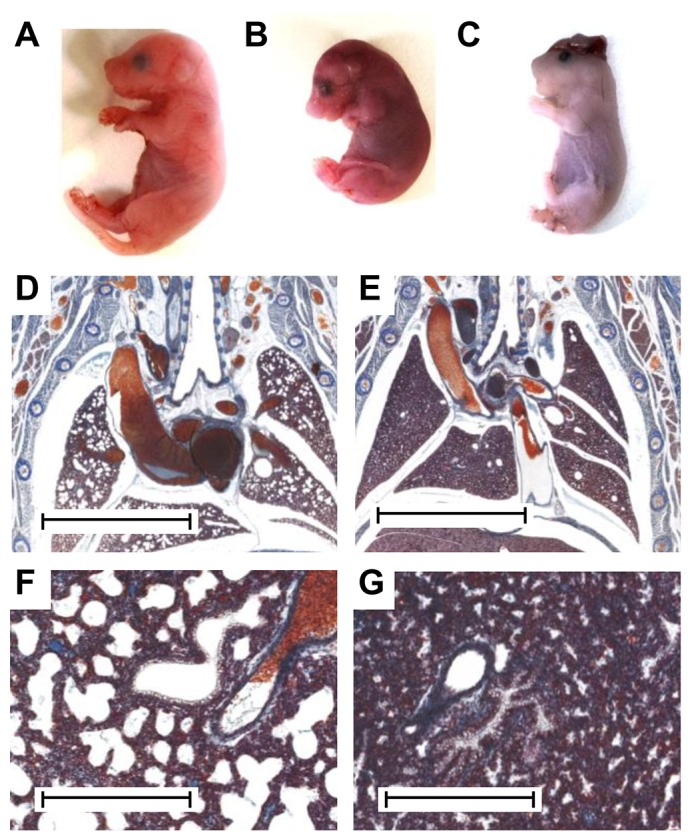
**Viability tests and anatomical characterization of foetuses at E18.5.** (A-C) Pictures of neonates, 30 min after caesarean section. Healthy foetuses moved and turned pink in the first few minutes after delivery. (A) Wild-type and (B) cyanotic Ms5Yah littermates. (C) A Ms5Yah foetus that presented craniorachischisis. (D-G) Anatomical characterization. Lungs from a wild-type foetus at low (D) and high magnifications (F) presented opened alveoli as the foetus was breathing before euthanasia. Lungs from a Ms5Yah foetus that was unable to breathe at low (E) and high (G) magnifications presented collapsed alveoli. Scale bars: 3 mm (D,E); 400 µm (F,G).

To determine whether Ms5Yah animals died *in utero* or postnatally, the foetuses of seven pregnant wild-type females that had been impregnated by Ms5Yah males were collected at embryonic day (E)18.5, a few hours before natural delivery. Over 52 foetuses were extracted by caesarean section and then analysed, and we genotyped 27 wild-type and 25 Ms5Yah foetuses, which is the normal genotypic Mendelian ratio. Although a majority of neonates, including all wild-type animals, turned pink and breathed actively (Fig. 1A), 40% (10/25 mice) of the Ms5Yah neonates showed severe respiratory distress, remained a cyanotic colour and died during the first 30 minutes after delivery (Fig. 1B). Two cases of craniorachischisis were also observed and genotyped as Ms5Yah (Fig. 1C). Although all 27 wild-type neonates survived without difficulty, only 52% (13/25 mice) of the mutant E18.5 neonates were able to breathe, although they were clearly underweight and needed more time to turn pink in comparison with wild-type littermates. Histological analysis revealed that the Ms5Yah foetuses that were unable to breathe appeared essentially normal compared to their wild-type littermates, with the exception of the lungs. Control littermates showed opened alveoli (Fig. 1D,F), whereas the intrapulmonary bronchi and alveoli of Ms5Yah stillbirths remained collapsed (Fig. 1E,G). However, thoracic muscles appeared to have developed normally, as did the thoraco-abdominal diaphragm muscle, larynx and extrapulmonar airway.

**Fig. 2.**
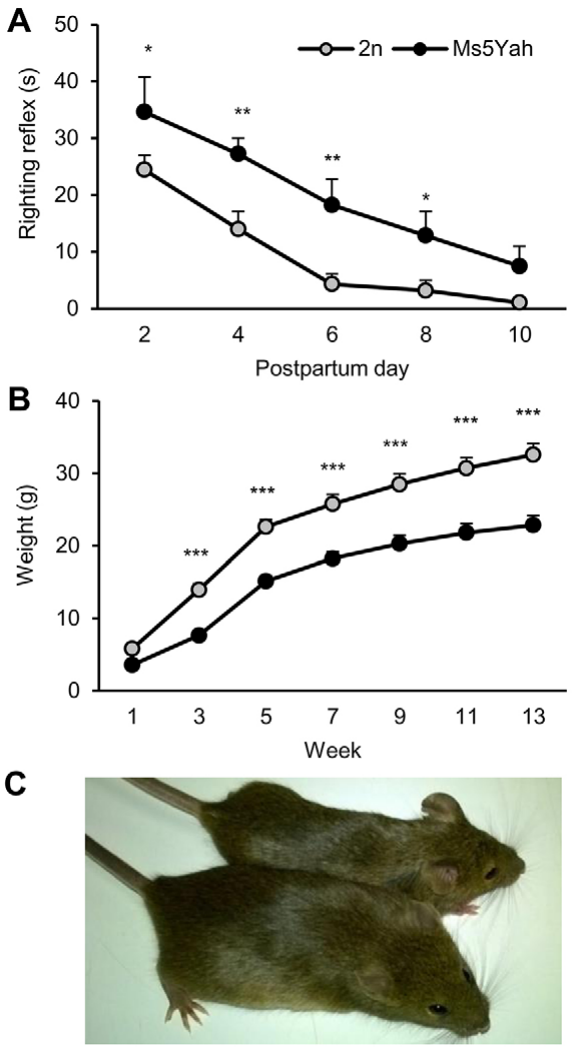
**Coordination capacities of newborn animals and development of their weight.** (A) Righting reflex of wild-type (2n) and Ms5Yah newborn mice. Graphs plotting the time (s) to recover natural posture when animals were placed on their back. (B) Evolution of body weight (g) from 1 to 13 weeks of age. The legend is the same as that shown in A. (C) 10-week-old wild-type (bottom of the image) and Ms5Yah (top of the image) littermates. All graphs depict the mean±s.e.m. *P<0.05, **P<0.01, ***P<0.001.

Surviving newborn mice were evaluated for the righting reflex from days 2 to 10 postpartum. When placed on the back, the latency to recover the natural posture was longer for Ms5Yah animals than for their wild-type littermates (repeated measures ANOVA ‘genotype’ *F*_(1,176)_=11.982, *P*=0.002; Fig. 2A). Body weights were recorded once a week (on the same day at the same time) from the age of 1 to 13 weeks. Ms5Yah mice were consistently underweight (repeated measures ANOVA ‘genotype’ *F*_(1,84)_=29.434, *P*<0.001; Fig. 2B) and smaller in size (Fig. 2C) compared with controls. Blood analyses conducted on adult animals revealed that Ms5Yah mice had a decrease in platelets (Ms5Yah, 774×10^3^±68 cells/µl; wild type, 1113×10^3^±79 cells/µl; ANOVA *F*_(1,17)_=10.361, *P=*0.005; supplementary material Table S1). We measured the bleeding time of animals and found that the time needed for wound coagulation was twice as long for Ms5Yah animals than it was for controls (Ms5Yah, 5.50±0.76 min; wild type, 2.99±0.33 min; ANOVA *F*_(1,20)_=9.086, *P=*0.007).

### Ms5Yah mice show motor coordination deficits without the alteration of exploration activity

The exploratory pattern and anxiety were evaluated in the open-field and elevated plus maze tests. In the open-field, no significant differences were observed between Ms5Yah and wild-type mice in either the distance travelled (ANOVA *F*_(1,22)_=0.677, *P=*0.420; supplementary material Fig. S1A) or the time spent in the centre area (Kruskal–Wallis test, H*=*0.121, *P*=0.728; supplementary material Fig. S1B). In the elevated plus maze, Ms5Yah mice explored the same number of arms (Kruskal–Wallis test, H*=*0.071, *P*=0.790; supplementary material Fig. S1C) and spent the same amount of time in open arms as the controls did (Kruskal–Wallis test, H*=*0.304, *P*=0.581; supplementary material Fig. S1D).

**Fig. 3.**
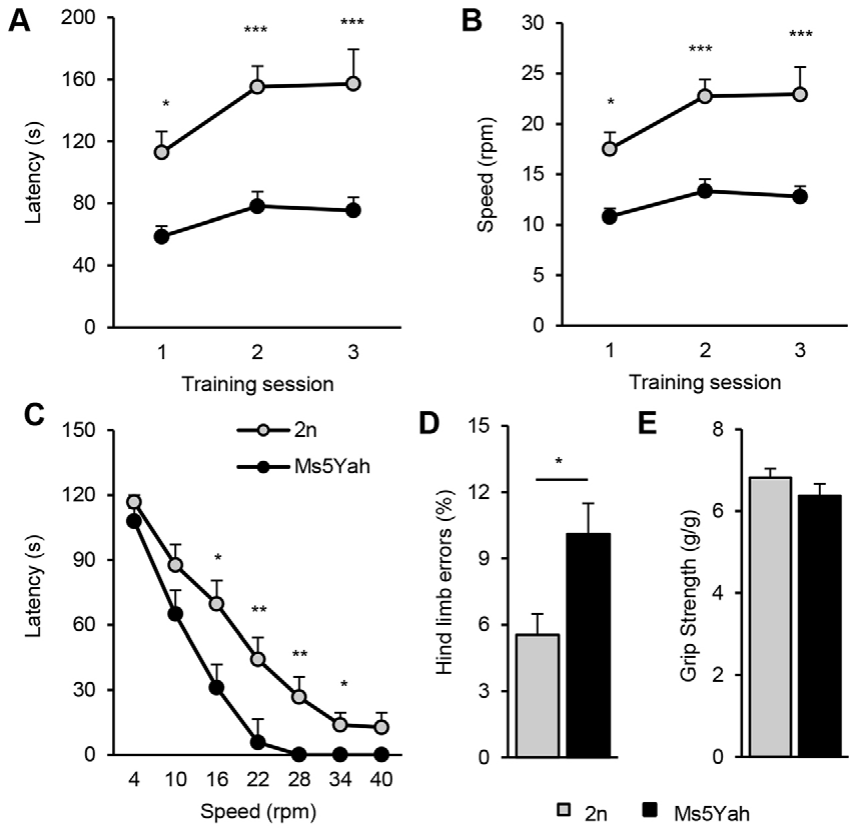
**Locomotor coordination and muscular capacities.** (A,B) Training phase of the rotarod test for wild-type (2n) and Ms5Yah mice. (A) Results are expressed as the time (s) that mice remained on an accelerating rod (4-40 rpm over 5 min) before falling. The legend given in C applies to panels A and B. (B) Mean rotational velocity (rpm) at the time of falling. (C) Test phase. The graphs plot the time (s) that mice stayed on the rod when tested at constant speeds between 4 and 40 rpm. (D) Notched bar test. The results are expressed as the percentage of hind paw errors made by mice when crossing the bar. (E) Four-paw grip test. These tests indicate that Ms5Yah show impaired motor coordination without alterations of muscular strength. All graphs depict the mean±s.e.m. *P<0.05, **P<0.01, ***P<0.001.

The rotarod test was performed for general motor coordination and balance evaluation. During the training period of three daily sessions, during which mice were placed on an accelerating rotarod, latencies to fall off the rod (Fig. 3A) and corresponding speed (Fig. 3B) were calculated. Compared with controls, Ms5Yah mice showed a global deficit in the ability to stay on the rod (repeated measures ANOVA ‘genotype’ *F*_(1,44)_=15.967, *P*<0.001). Wild-type animals were able to improve their coordination capacities from day 1 to day 3, whereas Ms5Yah presented impairments in staying on the rod on the first day and showed no amelioration on the third day (Tukey's post-hoc analysis, wild type on day 1 vs day 3, *q*=6.029, *P*<0.001; Ms5Yah day 1 vs day 3, *q*=2.085, *P=*0.313). The phase test comprised six trials of 2 min at constant speed, with the speed increasing between each trial [4, 10, 16, 22, 28, 34 and 40 rotations per minute (rpm)]. Two sessions were conducted on the same day. As for training, Ms5Yah mice showed difficulties in staying on the rod and exhibited a significant difference compared with controls from speeds 16 to 34 rpm (Kruskal–Wallis test, 16 rpm, H*=*3.889, *P*=0.049; 22 rpm, H*=*8.159, *P*=0.004; 28 rpm, H*=*9.255, *P*=0.002; 34 rpm, H*=*6.317, *P*=0.012; Fig. 3C). The notched bar test was used to evaluate hind-limb coordination. Animals had to cross a notched bar 20 times, and every time that the back paw went through the gap was counted as an error. Ms5Yah mice committed more errors than wild-type mice did (Kruskal–Wallis test, H*=*4.678, *P*=0.031; Fig. 3D). We then evaluated the muscle strength of Ms5Yah mice using the grip test. The strength index was calculated by the ratio of the force divided by the body weight. No difference was observed between mutants and controls (ANOVA *F*_(1,22)_=1.411, *P=*0.247; Fig. 3E). Histological analysis did not reveal any changes in the anatomy or thickness of the cerebellum (supplementary material Fig. S2A,B). All layers of mutant cerebella, including the molecular, granular and Purkinje cell layers, appeared normal (supplementary material Fig. S2C,D).

### Loss of one copy of the *App-Runx1* affects spatial learning and memory

Working memory was evaluated by recording spontaneous alternation in the Y-maze test. No difference in the number of arm entries (ANOVA *F*_(1,22)_=2.125, *P=*0.159; supplementary material Fig. S1E) or in the percentage of alternation was found (ANOVA *F*_(1,22)_=0.189, *P=*0.668; supplementary material Fig. S1F). We then assessed the recognition memory of mice using the novel object recognition (NOR) test. The choice to explore the novel object reflects the use of learning and recognition memory processes. Mice were placed in the open-field arena for 10 min with an object. Interestingly, Ms5Yah mice showed a higher exploration time than did controls (Ms5Yah, 12.7±1.2 s; wt, 9.2±0.9 s; ANOVA *F*_(1,17)_=4.776, *P=*0.043; supplementary material Fig. S1G). One hour after the first session, mice were placed in the same arena with the first object (considered as ‘known’) and a novel object. Both genotypes had similar exploration levels for the former object (ANOVA *F*_(1,17)_=1.695, *P=*0.210) and the new object (ANOVA *F*_(1,17)_=3.336, *P=*0.085), and showed similar discrimination indexes (ANOVA *F*_(1,17)_=0.415, *P=*0.528; supplementary material Fig. S1H), indicating no alteration of object recognition memory.

**Fig. 4.**
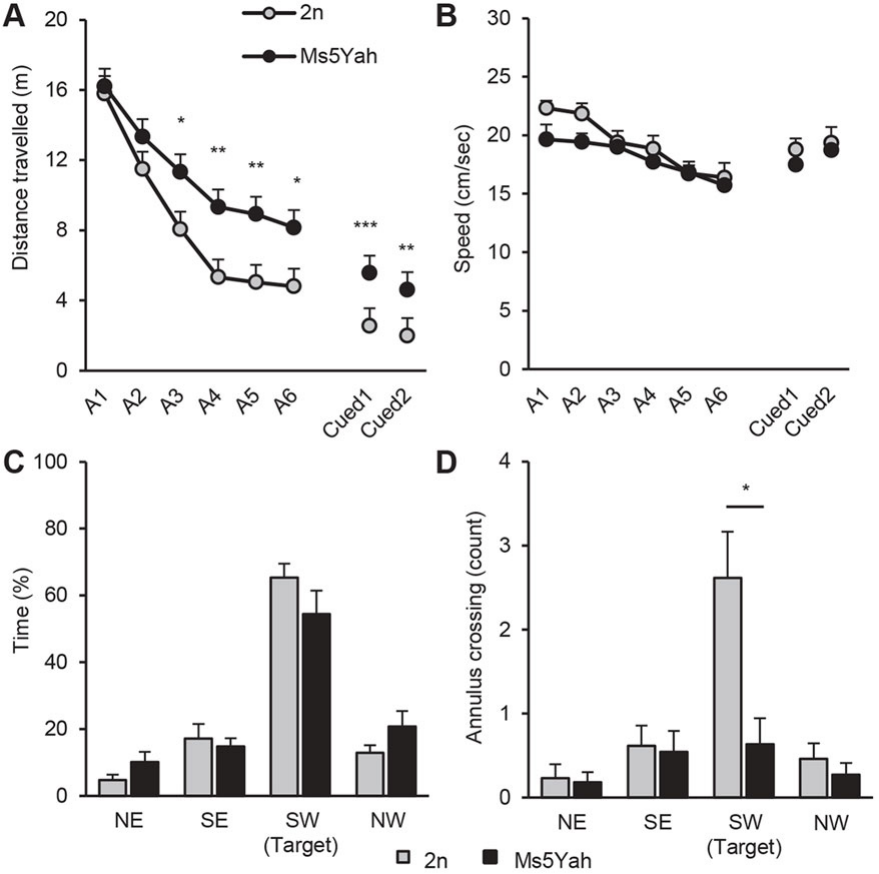
**Spatial learning and memory performances in the Morris water maze test.** (A) Distance travelled to find the platform along acquisition (A1–A6) and reversal (Cued1 and Cued2) sessions for wild-type (2n) and Ms5Yah mice. (B) Corresponding swimming speeds. (C,D) Removal session. Mice were scored for the percentage of time spent in the different quadrants (NE, north east; SE, south east; SW, south west; NW, north west) of the Morris water maze (C) and the annulus crossing (D) for one single trial where the platform was removed. Ms5Yah mice showed delays in the acquisition of the task and travelled a longer distance to find the platform. In addition to spatial learning impairments, the removal session indicated a deficit in accurate spatial memory as Ms5Yah mice had difficulty in remembering the exact position of the platform. All graphs depict the mean±s.e.m. *P<0.05, **P<0.01, ***P<0.001.

We further investigated spatial learning and memory in the Morris water maze. In this task, animals learn to locate the position of a submerged platform by using extra-maze spatial cues. In the acquisition phase, both genotypes were able to increase their capacities and travelled a shorter distance to find the platform between day 1 and day 6, indicating that the mice were able to learn (repeated measures ANOVA ‘day’, Tukey's post-hoc analysis; *F*_(1,110)_=25.709, *P*<0.001; wild type day 1 vs day 6, *q*=10.963, *P*<0.001; Ms5Yah day 1 vs day 6, *q*=7.400, *P*<0.001; Fig. 4A). However, Ms5Yah mice showed a global delay in the acquisition of the task and travelled a greater distance than controls to find the platform, with significant differences from day 3 to day 6 (repeated measures ANOVA ‘genotype’, Tukey's post-hoc analysis; *F*_(1,110)_=18.333, *P*<0.001; day 3, *q*=3.077, *P*=0.030; day 4, *q*=3.771, *P*=0.008; day 5, *q*=3.661, *P*=0.010; day 6, *q*=3.156, *P*=0.026). No difference in swimming speed was detected (repeated measures ANOVA ‘genotype’, *F*_(1,110)_=2.633, *P*=0.119; Fig. 4B), indicating normal coordination in a water environment for mutant mice. A probe test was performed on day 7. The platform was removed, and a unique trial was conducted to examine the duration spent in the target quadrant (Fig. 4, SO) and the number of times that mice crossed the exact spatial position of the platform (annulus crossing). Both genotypes spent similar amounts of time in the quadrant of interest (ANOVA *F*_(1,22)_=1.895, *P*=0.182; Fig. 4C), but Ms5Yah mice showed a clear diminution of annulus crossing (ANOVA *F*_(1,22)_=8.910, *P*=0.007; Fig. 4D), indicating a deficit in spatial memory. In the reversal sessions conducted on days 12 and 13, animals had to learn the new positions of a platform, which were made visible by an indicator. Mice from both groups needed a shorter amount of time to find the flagged platform than that required to find the hidden version, but Ms5Yah still showed a delay compared with controls (repeated measures ANOVA ‘genotype’, Tukey's post-hoc analysis; *F*_(1,22)_=28.753, *P*<0.001; day 12, *q*=6.267, *P*<0.001; day 13, *q*=5.462, *P*<0.001; Fig. 4A). When we examined the time spent in the learning target quadrant before mice reached the flagged platform, we observed a clear increase for Ms5Yah mice at day 2 (Ms5Yah, 9.3±1.0 s; wt, 6.4±0.8 s; Kruskal–Wallis test, H*=*7.573, *P*=0.006) and day 13 (Ms5Yah, 7.4±0.5 s; wild type, 4.8±0.5 s; Kruskal–Wallis test, H*=*11.487, *P*<0.001). This result indicates that mutant mice show difficulties in switching from a defined task to a new one.

In the optomotor response test, performed in the same lighting conditions as the Morris water maze test, both wild-type and Ms5Yah mice tracked rotating stripes for spatial frequencies of 0.13, 0.51 and 0.25 cycles/degree, but they failed to respond at 1.25 cycles/degree, suggesting that wild-type and Ms5Yah mice have comparable visual acuity that is sufficient to detect the flag placement on the Morris water maze platform.

### Transcriptomic expression analysis

**Fig. 5.**
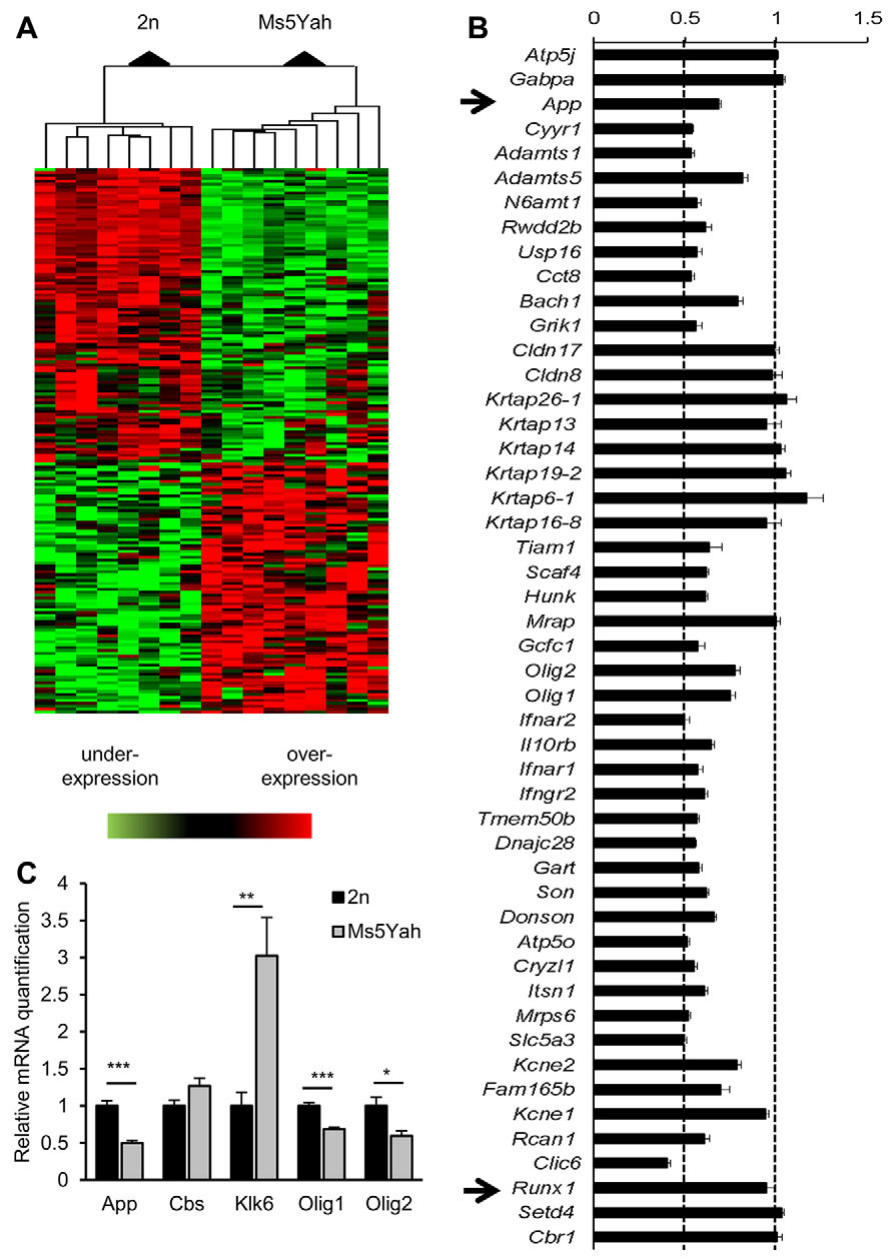
**Microarray and quantitative RT-PCR expression analyses on hippocampi.** (A) Clustering derived from statistically deregulated genes in Ms5Yah in comparison to expression in wild-type (2n) mice. Underexpression and overexpression are represented in green and red, respectively, and expression levels were calculated by comparison with the mean expression level of all arrays for each gene. (B) Expression profile of *App-Runx1* genes referenced on chips. Most of the genes had a fold change ranging from 0.5 to 0.8. Some genes of the *Krtap* cluster presented a fold change above threshold, which was probably due to several Krtap RNAs hybridizing to the same probes. (C) Quantitative PCR expression study focusing on candidate genes for memory deficits of Ms5Yah animals. The results confirm the transcriptome analysis showing the underexpression of *App*, *Olig1* and *Olig2* genes of the *App-Runx1* region, and the overexpression of *Klk6*. All graphs depict the mean±s.e.m. *P<0.05, **P<0.01, ***P<0.001.

Whole-genome expression arrays were performed on adult mice hippocampi to determine dosage-sensitive genes and to discern the deregulations on the whole genome using Affymetrix Gene Chip technology. Of the 23,332 probe sets, 12,870 were found to be expressed in the hippocampi. After normalization, we obtained a subset of 192 deregulated genes with a fold change higher than 1.2 or lower than 0.8 (Table 1). Clustering analysis revealed two distinct groups with respect to genotype-associated expression patterns (Fig. 5A). The first group, which comprised 96 genes that were downregulated in Ms5Yah mice, included 33 of the 45 transcripts of the *App-Runx1* region referenced on chips (Fig. 5B). As expected, *App* (fold change=0.68, *P*<0.001), *Olig1* (fold change=0.75, *P*<0.001) and *Olig2* (fold change=0.78, *P*<0.001) from the monosomic interval were found to be underexpressed (Fig. 5B). The second group contained 96 upregulated genes that were dispersed throughout the genome, including *Cbs* (fold change=1.34, *P*<0.001), which is a strong candidate gene for Down syndrome cognitive traits. In our analysis, a majority of deregulated genes showed a fold change that varied from 0.4 to 1.6, except for two genes, *Klk6* (fold change=1.70, *P*<0.001) and *Myoc* (fold change=2.49, *P*<0.001). We analysed the enrichment of functional annotation using the DAVID software (Huang et al., 2009b). Table 2 describes the principal entities affected by *App-Runx1* deletion. We found an enrichment of cell adhesion transcripts encoded by *App*, *Cntnap5b*, *Lgals3bp*, *Mag*, *Mcam*, *Npnt*, *Pcdhb2*, *Pcdhb3*, *Pcdhb4*, *Pcdhb6*, *Pcdhb7*, *Pcdhb8*, *Pcdhb16* and *Vwf* (fold enrichment=3.82; *P*=7.8×10^−5^; false discovery rate=0.099). Concerning molecular functions, the analysis pinpointed a clear enrichment for protocadherins (*Pcdhb2*, *Pcdhb3*, *Pcdhb4*, *Pcdhb6*, *Pcdhb7*, *Pcdhb8*, *Pcdhb16*; fold enrichment=35.8; *P*=2.9×10^−8^; false discovery rate=4.1×10^−5^) and for Ca^2+^ binding (*Efhd1*, *Itsn1*, *Npnt*, *Pcdhb2*, *Pcdhb3*, *Pcdhb4*, *Pcdhb6*, *Pcdhb7*, *Pcdhb8*, *Pcdhb16*, *Pla2g5*, *Pnck*, *Pon3*, *Ppp2r3a*, *Slc24a4*, *Smoc2*, *Sulf1*; fold enrichment=2.08; *P*=7.1×10^−3^; false discovery rate=8.73). Finally, we performed quantitative real-time (RT-)PCR to evaluate the level of expression of a few genes, including *App*, *Cbs*, *Klk6*, *Mag*, *Olig1* and *Olig2*. As shown in Fig. 5C, we found similar changes, except in the *Cbs* mRNA levels, which were not significantly different in control and Ms5Yah hippocampi.

**Table 1.**
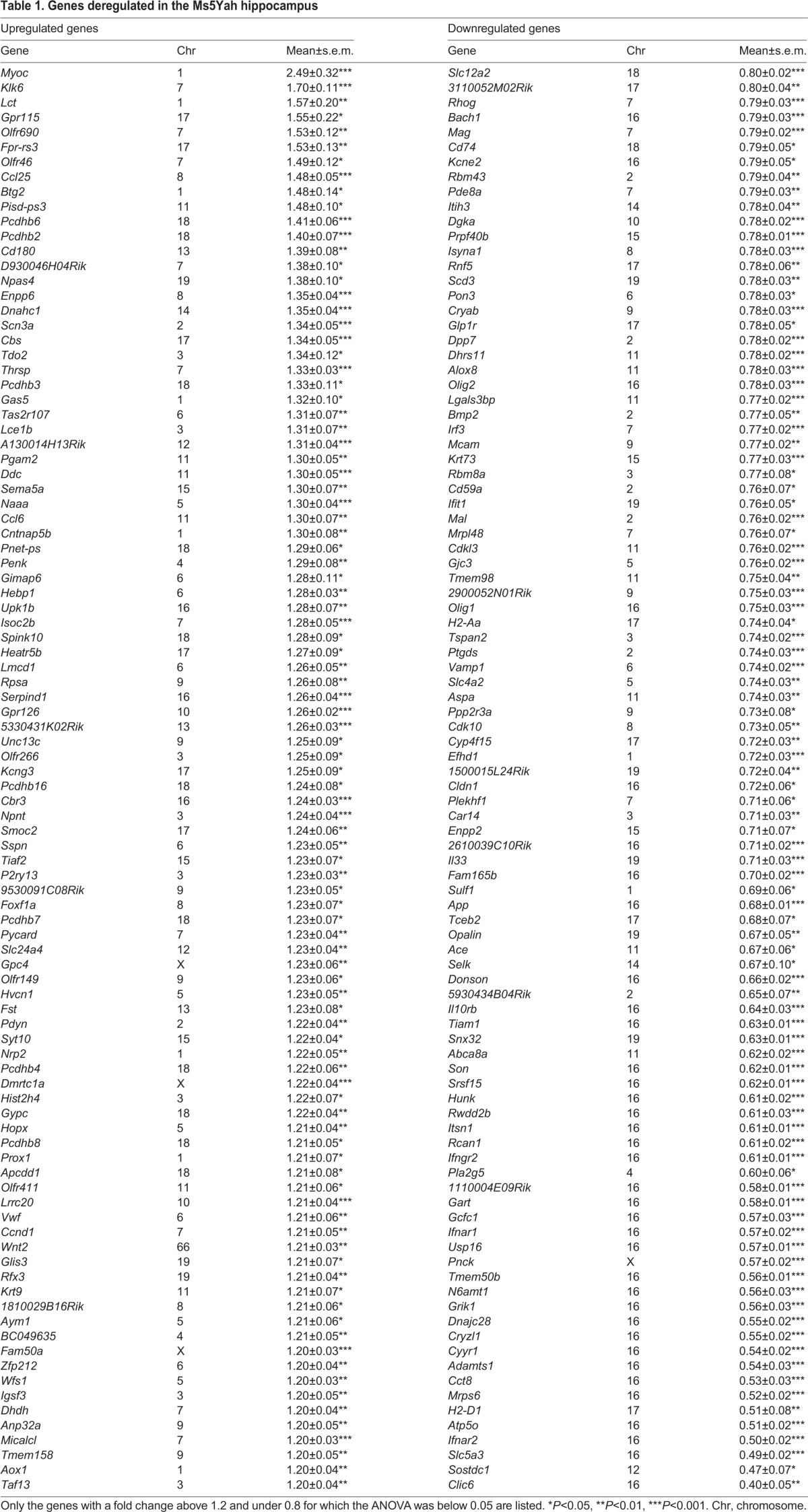
**Genes deregulated in the Ms5Yah hippocampus**

**Table 2.**
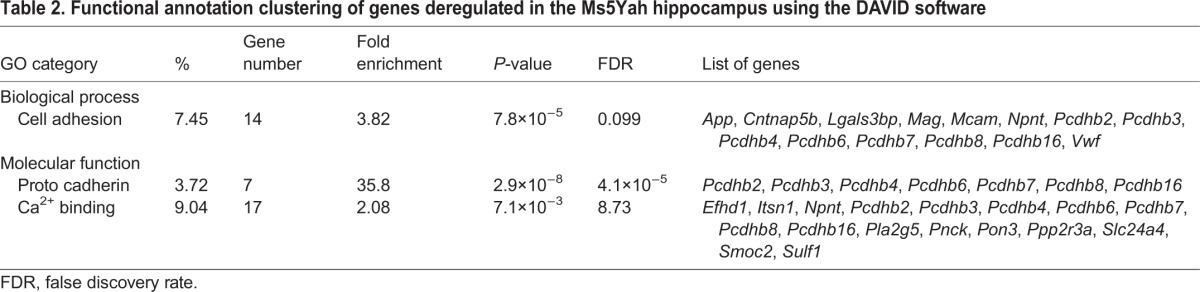
**Functional annotation clustering of genes deregulated in the Ms5Yah hippocampus using the DAVID software**

## DISCUSSION

Here, we report the characterization of the Ms5Yah mouse model that is monosomic for the 7.7-Mb *App–Runx1* region. This genetic interval overlaps approximately 50 known protein-coding genes that are located on Mmu16 and are homologous to the Hsa21q21.3-22.11 genes. Previous studies of monosomic mice for Mmu17 and Mmu10 syntenic regions corresponding to the telomeric part of Hsa21q have not revealed any morphological abnormalities and have shown only mild behavioural phenotypes (Besson et al., 2007; Duchon et al., 2011; Sahún et al., 2014; Yu et al., 2010). Two mouse models deleted in the Mmu16 syntenic region of Hsa21 have been characterized. Ms1Rhr mice deleted in the Hsa21q22.2 homologous region show brain volume alterations (Olson et al., 2007), and Ms1Dja mice deleted in the centromeric Hsa21q11.2-q21 homologous region present increased fat deposition (Migdalska et al., 2012). All of these mouse models present relatively incomplete phenotypes with regards to the severity of PM21 human symptoms. Conversely, the phenotypic analysis of Ms5Yah mice revealed dramatic changes in viability, morphology, motor coordination and cognition.

Ms5Yah mice were not viable on a pure C57BL/6N genetic background. The line was maintained on an F1 B6C3B genetic background to improve the survival of newborns. Viability tests revealed a few cases of craniorachischisis and a clear breathing incapacity for 40% of Ms5Yah neonates. Histological evaluation failed to reveal substantial malformations in any organ of the Ms5Yah foetuses that died. The only abnormality observed was pulmonary collapse, indicating an inability to breathe. The surviving animals were underweight and had coordination deficits. When put on their back, Ms5Yah pups needed more time to recover their normal position. This impairment in locomotion could limit the feeding capacities of pups and could potentially worsen the size deficit already observed at birth. With a longer bleeding time and decreased level of platelets, these deficits could explain why only 22.4% of animals that survived at weaning carried the *App-Runx1* deletion. In other analyses of the homologous regions covering the long arm of Hsa21 in mice (Besson et al., 2007; Duchon et al., 2011; Migdalska et al., 2012; Olson et al., 2007; Sahún et al., 2014; Yu et al., 2010), the *App-Runx1* interval corresponded to one crucial region of Hsa21 and contributed to embryonic growth, neural tube formation and lung function, at least at birth. We did not estimate lung function in adults; nevertheless, our data reinforce the hypothesis of lethality owing to full monosomy 21 in humans (Schinzel, 1976; Toral-Lopez et al., 2012).

Adult behavioural phenotyping confirmed dramatic changes in locomotor coordination and spatial learning, as well as memory defects of Ms5Yah mice. During the open-field and elevated plus maze tests, Ms5Yah mice showed normal exploratory behaviour and did not demonstrate anxiety. In the rotarod test, mutant mice showed severe deficits in motor skills. Ms5Yah animals were unable to improve their performances during the training phase and failed to stay on the rod, even at speeds lower than those used in the test phase. Coordination deficits were confirmed by using the notched bar test. The grip-strength test did not reveal any muscular weakness, and no gross anatomical changes were observed upon histological analysis of Ms5Yah cerebellum (supplementary material Fig. S2). These data all pinpoint a motor coordination deficit of mutant mice without alterations of muscular strength and general activity. The Y-maze and novel object recognition tests revealed no perturbation of short-term memory. In the water-maze test, Ms5Yah animals showed a global delay in the acquisition of the task and travelled a greater distance to find the platform without changing their swimming speed. In the removal session, mutant mice spent the same amount of time in the target quadrant as controls did, but the mutants showed difficulty in remembering the exact position of the platform, which indicates an impairment in spatial memory. Finally, during the reversal sessions, mutant mice showed difficulty in switching from a defined task to a new one. The entorhinal cortex is known to integrate sensory information in several cortical areas and then transfer them to the hippocampus. Globally, Ms5Yah mice showed a deficit of spatial learning and memory without impact on recognition and working memory recognition, suggesting that the hippocampus is not strongly affected and that the defect might depend on the entorhinal cortex or alterations in the entorhino-hippocampal circuitry (Sasaki et al., 2015). Moreover, the medial entorhinal cortex and the parahippocampal cortex, with their specialization for spatial computing and contextual information, might be more greatly affected than the lateral entorhinal cortex and the perirhinal cortex, which are more specialized in object and object-place information (Eichenbaum et al., 2012; Sauvage et al., 2010). Additional experiments will be needed to decipher this hypothesis.

Hippocampus-dependent spatial learning and locomotor learning deficits in mice can be directly related to intellectual disabilities of PM21 individuals. To evaluate the impact of the reduction of *App-Runx1* gene dosage on the whole genome, we performed transcriptomic expression analysis on the hippocampus. After enhancing the stringency and power of the statistical tests, the analysis highlighted 192 deregulated protein-coding genes, half of which were downregulated and the other half of which were upregulated. Functional annotation analysis revealed an enrichment of the genes related to cell adhesion pathways (*App*, *Cntnap5b*, *Lgals3bp*, *Mag*, *Mcam*, *Npnt*, *Pcdhb2*, *Pcdhb3*, *Pcdhb4*, *Pcdhb6*, *Pcdhb7*, *Pcdhb8*, *Pcdhb16* and *Vwf*). An important number of protocadherins, which represent part of a gene cluster that is located on Mmu18, appeared to be upregulated in Ms5Yah hippocampi. Some of these cell-cell adhesion proteins are involved in the intracellular signalling pathways that are associated with neuropsychiatric disorders and cognitive impairments (Redies et al., 2012). Notably Pcdhb6, Pcdhb8, Pcdhb9 and Pcdhb10 are found expressed in the hippocampus and the entorhinal cortex in rats (Bekirov et al., 2002). An interesting hypothesis would be that the overexpression of these cell-adhesion molecules would negatively impact the medial entorhinal cortex, the parahippocampal cortex or the entorhino-hippocampal circuitry. *De novo* mutations in *Pcdhb4*, which was found in our deregulated list, have been associated with sporadic autism spectrum disorders (O'Roak et al., 2012). Of the 45 genes of the *App-Runx1* region that were referenced on chips, 33 were found to be downregulated, including *App*, which has been implicated in many neuronal processes, such as synaptogenesis (Wang et al., 2009), neural plasticity and memory (Turner et al., 2003). Little is known regarding heterozygotes, but *App*-deficient mice are viable and fertile, and present a 15-20% decrease in weight, reduced forelimb grip strength and impaired locomotor activity (Zheng et al., 1995), which is highly similar to the Ms5Yah phenotypes. Aged *App*-knockout mice also show impairments in learning and memory that are associated with a deficit in hippocampal long-term potentiation (Ring et al., 2007). The two basic helix-loop-helix oligodendrocyte transcription factors *Olig1* and *Olig2* were also found to be downregulated. These genes are implicated in the generation of motoneurons and oligodendrocytes (Lu et al., 2002). *Olig1* and *Olig2* triplication is associated with developmental brain defects in Down syndrome (Chakrabarti et al., 2010). In *Olig1*- and *Olig2*-deficient mice, motoneurons are converted to V2 interneurons in the spinal cord, and oligodendrocytes fail to differentiate throughout the nervous system (Zhou and Anderson, 2002). The underexpression of *Olig1* and *Olig2* might play a role in the abnormal motor coordination observed in Ms5Yah animals. Our transcriptomic study revealed an important upregulation of the *Klk6* gene that encodes kallikrein-related peptidase 6, which is a biomarker of Alzheimer's disease (Diamandis et al., 2000). Interestingly, *in vitro* substrates of this serine protease include the amyloid precursor protein (Ashby et al., 2010), which reinforces the idea that *App* is an interesting candidate gene for Ms5Yah phenotypes. Nevertheless, proteomic analyses should be performed to confirm the effects of *App-Runx1* deletion.

Overall, the results in mice presented here confirm the crucial importance of the *APP-RUNX1* region in partial monosomy 21 (Chettouh et al., 1995; Lyle et al., 2009). Human PM21 individuals present postnatal growth retardation, short stature, craniofacial malformations, psychomotor retardation and intellectual disability, and Ms5Yah mice show developmental delay, reduction of size and weight, thrombocytopenia, motor coordination deficits, and spatial learning and memory impairments. To date, no human carrying a deletion for the entire *APP-RUNX1* region has been described in the literature, indicating that this monosomic state is possibly not compatible with viability. A meta-analysis of PM21 cases has revealed that individuals carrying large deletions from the centromere to approximately 31.2 Mb of the long arm of Hsa21 show severe phenotypes (Roberson et al., 2011). A majority of these large deletions encompass the *APP-SOD1* region. Fewer than five individuals carrying partial deletions in the *SOD1-RUNX1* region have been identified. By combining information from previous studies, a 0.56-Mb crucial region has been identified that contains four genes – *KCNE1*, *RCAN1*, *CLIC6* and *RUNX1* – which are associated with severe congenital heart defects (Click et al., 2011; Lindstrand et al., 2010; Lyle et al., 2009). PM21 deletions encompassing *RUNX1* (Shinawi et al., 2008) and *RUNX1* haplodeficiency (Jalagadugula et al., 2010; Liew and Owen, 2011) are also commonly associated with thrombocytopenia, which is consistent with our haematology analysis. By using genetic approaches to restore the expression of genes included in the *App-Runx1* region, we think that our mouse model will aid in the identification of candidate genes responsible for partial monosomy 21 symptoms and in the development of therapies.

## MATERIALS AND METHODS

### Mouse lines, genotyping and ethics statement

Del(16*App-Runx1*)5Yah mice, also named Ms5Yah, were kept and bred on an F1 B6C3B background. The model was generated through Cre-LoxP *in vivo* recombination (Brault et al., 2006) using a mouse line carrying two loxP sites inserted at *App* and *Runx1* in a cis configuration, as described previously (Raveau et al., 2012). The Ms5Yah allele was identified by using PCR analysis with one forward primer (5′-ATCCGGGAATGGT-CCCTA-3′) specific for the wild-type allele, one forward primer (5′-CAAGCACTGGCTATGCATGT-3′) specific for the Ms5Yah allele and a Ms5Yah/wild-type reverse primer (5′-GTTCGTTGCCTGAAGGAGAG-3′) common to both alleles. PCR analyses gave wild-type and Ms5Yah products of 482 bp and 328 bp, respectively. The experimental procedures were approved by the local ethical committee Com'Eth under accreditation number 2012-069 with Y.H. as the principal investigator in this study (accreditation 67-369).

### Viability and early postnatal tests

Viability tests were conducted in order to study causes of early postnatal lethality. Approximately 52 foetuses at E18.5 were isolated from seven pregnant females, 1 day before natural delivery, to monitor their capacity to survive at birth. Foetuses were placed on a warm plate at 37°C and rolled gently to stimulate them to breathe. At 30 min after extraction, the numbers of pink and moving animals versus cyanotic animals that were unable to breathe were counted. Tail samples were collected for genotyping.

Litters of four females, including eight Ms5Yah and 16 wild-type animals, were evaluated for the righting reflex. Briefly, animals were put on their back, and the time needed to recover their posture was measured. The test was conducted every day from postpartum day (P)2 to P10. To study the weight development, body weights were recorded once a week (same day at the same time) from the age of 1 to 13 weeks.

### Behavioural analysis

A series of behavioural experiments was conducted to evaluate activity, memory and locomotor coordination of mice. The majority of the experimental procedures have been described previously (Duchon et al., 2011). To produce experimental groups, only animals coming from litters containing a minimum of two male pups were selected. A cohort that included 11 Ms5Yah and 13 control mice was generated. After weaning, animals were gathered by litters in a 39×20×16-cm cage (Green Line, Techniplast, Italy), where they had free access to water and food (D04 chow diet, Safe). The temperature was maintained at 23±1°C, and the light cycle was controlled on a 12-h light and 12-h dark cycle. Mice were transferred from the animal facility to the phenotyping area at the age of 12 weeks and were screened for behavioural tests from 14 to 19 weeks. Animals were transferred to experimental room antechambers 30 min before each experiment. All experiments were conducted between 08:00 and 13:00. Tests were conducted in the following order: elevated plus maze (at age 14 weeks), open field (at age 14 weeks), novel object recognition (at age 15 weeks), Y maze (at age 15 weeks), rotarod (at age 16 weeks), notched bar (at age 17 weeks), grip strength (at age 17 weeks) and Morris water maze (at age 18-19 weeks).

The elevated plus maze allows for the evaluation of anxiety. The apparatus comprised two opposed open arms (30×5 cm) that were crossed by two enclosed arms (30×5×15 cm) and elevated 66 cm from the floor. The light intensity at the extremity of the open arms was kept at 50 lux. Each mouse was tested for 5 min after being placed in the central platform and was allowed to freely explore the apparatus. The number of entries and the time spent in the open arms were used as an index of anxiety. Closed-arm entries and the number of rears in the closed arms were used as measures of general motor activity.

The open-field test was used to evaluate exploration behaviour. Mice were tested in automated open fields (44.3×44.3×16.8 cm) made of PVC with transparent walls and a black floor, and covered with translucent PVC (Panlab, Barcelona, Spain). The open-field arena was divided into central and peripheral regions, and was homogeneously illuminated at 150 lux. Each mouse was placed in the periphery of the open field and allowed to freely explore the apparatus for 30 min. The distance travelled, the number of rears, and the time spent in the central and peripheral regions were recorded over the test session.

The novel object recognition test is based on the innate tendency of rodents to differentially explore novel objects over familiar ones. On the first day, mice were habituated to the open-field arena for 30 min under 60 lux. On the following day, animals were submitted to the first 10-min trial, during which they were individually placed in the open field at 60 lux in the presence of object A (marble or dice), which was placed at 10 cm from one corner of the box. The exploration time of object A (when the animal's snout was directed towards the object at a distance ≤1 cm) was recorded. A 10-min retention trial (second trial) was tested 1 h later. The former object (object A) and the novel object (object B) were placed at 10 cm from two open-field corners (the distance between the objects was approximately 20 cm), and the exploration time of the two objects was recorded. A discrimination index was defined as (*t*_B_/(*t*_A_+*t*_B_))×100. All mice that did not explore the first object for more than 3 s were excluded from the analysis.

The Y-maze test is used to evaluate short-term working memory. This test is based on the innate preference of animals to explore an arm that has not been previously explored, a behaviour that, if it occurs with a frequency of greater than 50%, is called spontaneous alternation. In this test, we used a Y-shaped maze with three white, opaque plexiglas arms of equivalent length forming a 120° angle with each other. The arms have walls with specific motifs that allow for their distinction from each other. After introduction at the centre of the maze (illuminated at 60 lux), animals were allowed to freely explore the three arms for 6 min. The number of arm entries and the number of triads were recorded to calculate the percentage of alternation.

The rotarod test assesses sensorimotor coordination and balance. The apparatus (Biosed, France) was a rotating bar that is 5 cm in diameter (hard plastic material covered by grey rubber foam) on which mice are placed facing the direction of rotation. Animals were ﬁrst habituated to stay on the rod for 30 s at a constant speed of 4 rpm. This was followed by three training days with four trials per day. Mice were placed on an accelerating rod increasing from 4 rpm to 40 rpm in 5 min under 100-lux lighting. The test was stopped when the mouse fell down from the rod or when there was more than one passive rotation. The latency to fall and the maximum speed before falling was recorded. On the fourth day, mice had two test sessions comprising six consecutive trials of 2 min at constant speed, with increasing speed between each trial (4, 10, 16, 22, 28, 34 and 40 rpm). For each trial, the latencies to fall off the rod were recorded. A maximum latency of 2 min was attributed for each animal that did not fall during the test.

The notched bar test was used to test the hindlimb coordination. Mice were trained with a bar that was a 1.7-mm-wide and 50-cm-long natural wooden piece bearing terminal platforms of 6 cm×6 cm. On the second day, mice were tested under 100-lux lighting with a notched bar of the same dimensions comprising 12 platforms of 2 cm^2^ spaced by 13 gaps of 2 cm^3^. Animals had to cross the notched bar twice for training and 15 times for the test. Every instance of a back paw going through the gap was considered an error, and the global error percentage was calculated.

The grip-strength test was used to evaluate muscular strength. Mice were first weighed and tested with a handy force gauge (Bioseb, France). Animals were placed on the instrument grid and pulled by the tail until they let go. The force (***g***) was related to animal weight (g).

The Morris water maze is a paradigm for spatial learning and memory. The water maze was a circular pool (150-cm diameter, 60-cm height) filled to a depth of 40 cm with water maintained at 20-22°C and made opaque using a white aqueous emulsion (Acusol OP 301 opacifier). An escape platform, 6 cm in diameter and made of rough plastic, was submerged 1 cm below the water surface. The test began with 6 days of acquisition, four trials per day, under 120 lux. Each trial started with the mice facing the interior wall of the pool and ended when animals climbed onto the platform or after a maximum searching time of 90 s. The platform was located at the same position for all the four trials, but the starting positions changed randomly between each trial with departures from each cardinal point. The distances travelled to find the platform and swimming speeds were analysed each day. On the 7th day, mice were given a single trial of 60 s during the probe test or a removal session in which the platform was removed. The distance travelled and the duration spent in each quadrant [north west (NW), north east (NE), south west (SW), south east (SE)] were recorded. The annulus crossing index was calculated as the number of times that animals crossed the exact platform position. On the 12th and 13th days, mice were given reversal sessions with four trials of 90 s per day. The platform was made visible by a small dark ball placed 12 cm on top of the platform, and the external cues were hidden by surrounding the pool with a black curtain. To ensure that the mouse used the platform cue, the starting position and platform position were changed for each trial.

### Optomotor response

Mice (three wild type and two Ms5Yah) were placed on a platform (11.5-cm diameter grid, 19.0 cm above the bottom) on the axis of a rotating drum (29.0 cm in diameter, 2 rpm), with the inside surface covered with alternating black and white stripes. The stripe spatial frequency was alternated at 0.25, 0.51 and 1.27 and 0.13 cycles/degree. The light intensity was adjusted to 120 lux, as in the Morris water maze test. Mice were recorded by using a digital video camera (Sony, DCR-TRV24E) that was placed above them. For each spatial frequency, the drum was rotated alternately clockwise and anticlockwise for 1 min or until two clear head-tracking movements were scored by two observers.

### Blood haematology and bleeding time

Blood was collected from 25-week-old males (11 Ms5Yah and 11 wild-type littermates) by retro orbital puncture under isoflurane anaesthesia at 00.00 (time 0) after 4 h of fasting. A total of 120 µl of blood was collected into EDTA-coated paediatric Microvette tubes for haematological analysis. A complete blood count, including total erythrocyte, leukocyte and platelet counts, differential leukocyte count (granulocytes: neutrophils, eosinophils and basophils, lymphocytes and monocytes), haemoglobin and haematocrit measurement, and calculation of blood indexes (mean cell volume, mean corpuscular haemoglobin concentration), was performed on total blood using an Advia 120 haematology analyser (Siemens).

To evaluate whether the platelet deficit of Ms5Yah mice influenced the coagulation process, bleeding time was measured in the same animals at 27 weeks of age. After 5-mm distal tips had been transected, the tail was immersed into PBS at 37°C. Bleeding time was measured from incision to the first time that the bleeding stopped.

### Histology

To evaluate the anatomy of the foetuses at E18.5, one Ms5Yah foetus that was unable to breathe, one Ms5Yah foetus with craniorachischisis and one wild-type foetus were fixed at the end of the viability test and subjected to histological analysis. Foetuses were decalcified in DC3 solution before being embedded in paraffin and cut into 7-µm-thick sections that were stained using modified Mallory's tetrachrome (hematoxylin, Acid Fuschin, Aniline Blue and Orange G).

For cerebellum histological and anatomical studies, five Ms5Yah and four control brains were collected, fixed in 10% formalin for 48 h and embedded in paraffin. Transversal sections (5-µm thick) through the middle of the cerebellum were taken and then stained with hematoxylin and eosin. Stained sections were digitalized using a slide scanner (Nanozoomer 2.0-HT, Hamamatsu, Japan), and measurements of the cerebellar vermis and hemisphere thickness relative to whole thickness were performed using the NDPview software of the digital scanner.

### Total RNA extraction

For Affymetrix arrays, hippocampi were isolated from Ms5Yah (*n*=9) and wild-type control (*n*=8) animals at 4 months and then flash frozen. Total RNA was prepared using TRIzol reagent (Invitrogen) and purified using the RNeasy Mini Kit (Quiagen) according to the manufacturer's instructions. Sample quality was checked using an Agilent 2100 Bioanalyzer (Agilent Technologies).

### Whole-genome expression arrays

In conjunction with the Affymetrix GeneChip WT Terminal Labeling Kit, the Ambion WT Expression Kits were designed to generate amplified and biotinylated sense-strand DNA targets from the entire expressed genome and hybridized onto GeneChip Mouse Exon 1.0ST arrays (Affymetrix). Chips were washed and scanned using the GeneChip Scanner 3000. Digitized images were generated with AGCC v3.2 (Affymetrix) software, and data files were generated with the Expression Console v1.1 (Affymetrix) software. The raw data were processed using the Robust Multiarray Average (RMA) algorithm (developed by Irizarry et al., 2003) and values that were log transformed using the Partek (Partek Inc.) and GeneSpring (Agilent Technologies) software. Statistical analysis was performed using GeneSpring (one-way ANOVA), and the 192 genes with a fold change above 1.2 or below 0.8 and a *P*-value below 0.05 were selected for clustering analysis. Hierarchical clustering was conducted using the Cluster3.0 software (de Hoon et al., 2004), implementing Euclidian distances to calculate the distances between the genes and between the samples. Calculated distances were then clustered by complete linkage clustering. Post-hoc analysis using GeneSpring gave a list of statistically deregulated genes. Known mammalian phenotype database (Mouse Genome informatics, The Jackson Laboratory) and functional annotation clustering using Database for Annotation, Visualization and Integrated Discovery (DAVv) bioinformatics were performed to estimate the potential impact of deregulated genes in transgenic mice. This latter tool primarily provides typical batch annotation and gene-gene-ontology (gene-GO)-term enrichment analysis to highlight the most relevant GO terms that are associated with a given gene list (Huang et al., 2009a). The microarray data have been deposited in the NCBI Gene Expression Omnibus (GEO; Edgar et al., 2002) and are accessible through GEO series accession number GSE58639.

### Quantitative RT-PCR analysis

For quantitative RT-PCR analyses, hippocampi were isolated from 3-month-old Ms5Yah (*n*=6) and wild-type control (*n*=7) animals, and were then flash frozen. cDNA synthesis was performed using the SuperScript III First-Strand Synthesis SuperMix for quantitative RT-PCR (Invitrogen). A series of primer pairs (available upon request) was designed to span the intron-exon junctions. Efficiencies of the TaqMan assays were checked using a cDNA dilution series from the extracts of hippocampus samples. The quantitative PCR was performed with 250 nM of each primer and 125 nM of high-performance liquid chromatography (HPLC)-purified fluorescein amidite (FAM)-labelled TaqMan probes in a final reaction of 15 µl with a standard amplification procedure. Normalization was performed by amplifying five housekeeping genes (*Actb*, *Pgk1*, *Hprt1*, *Ppia* and *Gnas*) in parallel and by using the GeNorm procedure (Vandesompele et al., 2002) to correct the variations in the amount of source RNA in the starting material. All sample tests were performed in triplicate.

### Statistical analysis

The results were processed for statistical analysis using the Sigma Plot software (Sigma). All acquired behavioural data were analysed using a one way ANOVA test when applicable or the non-parametric Kruskal–Wallis test. Post-hoc analysis was performed using Tukey's test. Pearson's Chi-squared test was used to evaluate mutant allele transmission. The data are represented as the mean±s.e.m., and the significant threshold was *P*<0.05 unless otherwise indicated.

## Supplementary Material

Supplementary Material
