# Supplementary material for: Deletion of the App-Runx1 region in mice models human partial monosomy 21
Source: Dis Model Mech. 2015 Jun 1;8(6):623–34. doi: 10.1242/dmm.017814 (PMC4457029; doi:10.1242/dmm.017814)
Supplement: Supplementary Material [file supp_8_6_623__index.html]

Deletion of the App-Runx1 region in mice models human partial monosomy 21 — Supplementary Material 

# Deletion of the *App-Runx1* region in mice models human partial monosomy 21

## DMM017814 Supplementary Material

**Files in this Data Supplement:**

- **Supplementary Material**
