# Supplementary material for: Deletion of the App-Runx1 region in mice models human partial monosomy 21
Source: Dis Model Mech. 2015 Jun 1;8(6):623–34. doi: 10.1242/dmm.017814 (PMC4457029; doi:10.1242/dmm.017814)
Supplement: Supplementary Material [file supp_8.6.623_DMM017814.pdf]

SUPPLEMENTARY MATERIALS AND FIGURES

Cell Type/Parameter	Unit	2n	Ms5Yah
White blood cells	cells/µL	$7.4 \times 10^3 \pm 1.03$	$5.4 \times 10^3 \pm 0.7$
Red blood cells	cells/µL	$9.0 \times 10^6 \pm 0.6$	$8.5 \times 10^6 \pm 0.7$
Hemoglobin	g/dL	13.0 ± 0.8	12.0 ± 1.0
Hematocrit	%	46.8 ± 2.6	42.5 ± 3.3
Platelets	cells/µL	$1113 \times 10^3 \pm 79$	$774 \times 10^3 \pm 68$ **
Lymphocytes	cells/µL	$5.1 \times 10^3 \pm 0.7$	$3.5 \times 10^3 \pm 0.4$
Monocytes	cells/µL	$0.20 \times 10^3 \pm 0.03$	$0.13 \times 10^3 \pm 0.03$
Neutrophil granulocytes	cells/µL	$1.9 \times 10^3 \pm 0.3$	$1.5 \times 10^3 \pm 0.3$
Eosinophil granulocytes	cells/µL	$0.27 \times 10^3 \pm 0.08$	$0.35 \times 10^3 \pm 0.12$
Basophil granulocytes	cells/µL	25.0 ± 5.2	17.8 ± 4.0
Large cells	cells/µL	63.0 ± 13.4	35.6 ± 3.8

Table S1. Blood hematology. Compared to controls, Ms5Yah adult mice show a significant decrease of platelets. Results are mean ± SEM. $^{**}p < 0.01$.

Supplementary Figure S1. Exploration activity and short-term memory performance. (**A-B**) Open field test. Results are expressed as mean distance travelled (m) (**A**), and time percentage spent in the central area (**B**) over a 30 min test. (**C-D**) Elevated plus maze test. Number of arm entries (**C**) and percentage of time spent in the open arms (**D**). No difference in anxiety and exploration pattern was noticed between Ms5Yah and controls. (**E-F**) Y maze test. Number of arm entries (**E**) and percentage of alternation (**F**) over a 6 min test. (**G-H**) Novel object recognition (NOR) test. (**G**) Exploration time (s) of the first object. (**H**) Discrimination index.

Data indicate that Ms5Yah mice present similar short-term memory capacities than controls. All graphs depict mean ± SEM. $^{*}p < 0.05$.

Supplementary Figure S2. Transversal sections of the cerebellum stained with hematoxylin and eosin. Comparison of wild-type (**A**, **C**) and Ms5Yah (**B**, **D**) sections at low (**A**, **B**, scale = 2 mm) and high magnification (**C**, **D**, scale = 200 µm) through the middle of the cerebellum revealed no anatomical abnormality. At the histological level, all layers of the mutant cerebellum including the molecular, granular and Purkinje cell layers appeared normal.

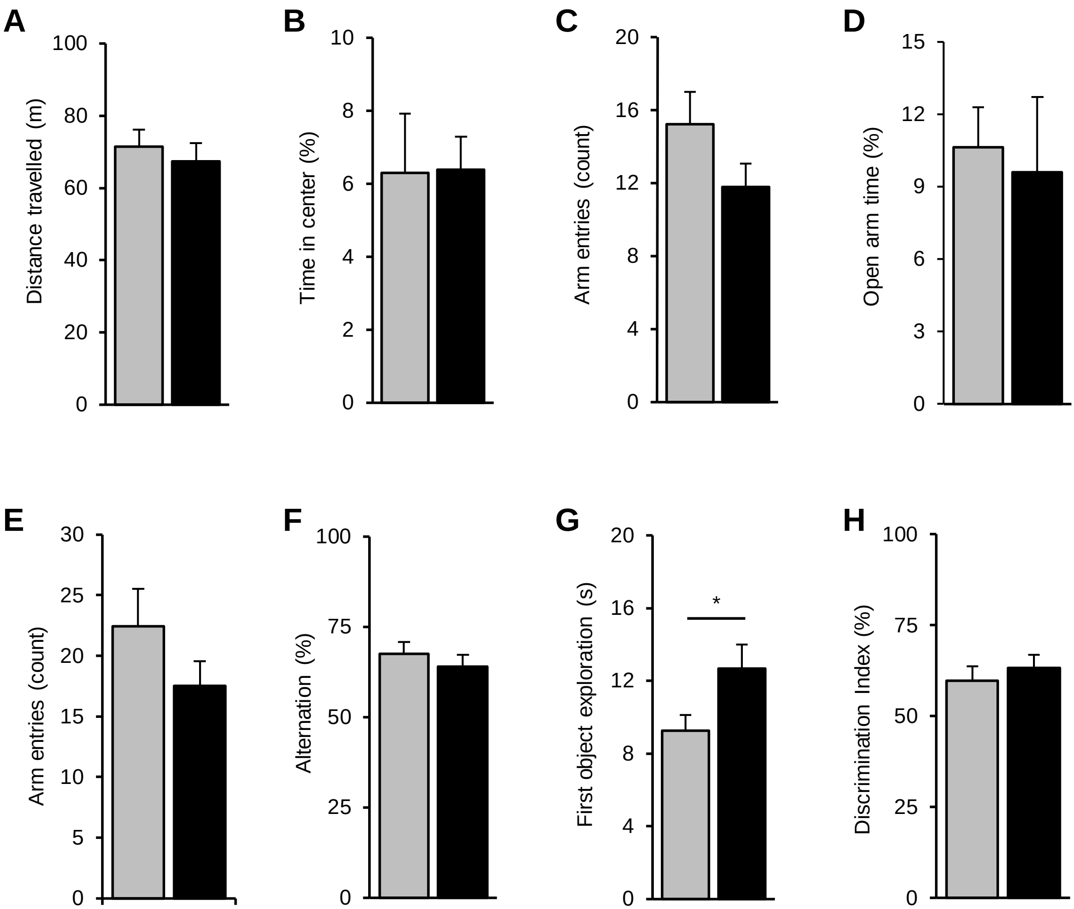
A
Distance travelled (m)
100
80
60
40
20
0
B
Time in center (%)
10
8
6
4
2
0
C
Arm entries (count)
20
16
12
8
4
0
D
Open arm time (%)
15
12
9
6
3
0
E
Arm entries (count)
30
25
20
15
10
5
0
F
Alternation (%)
100
75
50
25
0
G
First object exploration (s)
20
16
12
8
4
0
*
H
Discrimination Index (%)
100
75
50
25
0

Figure S1

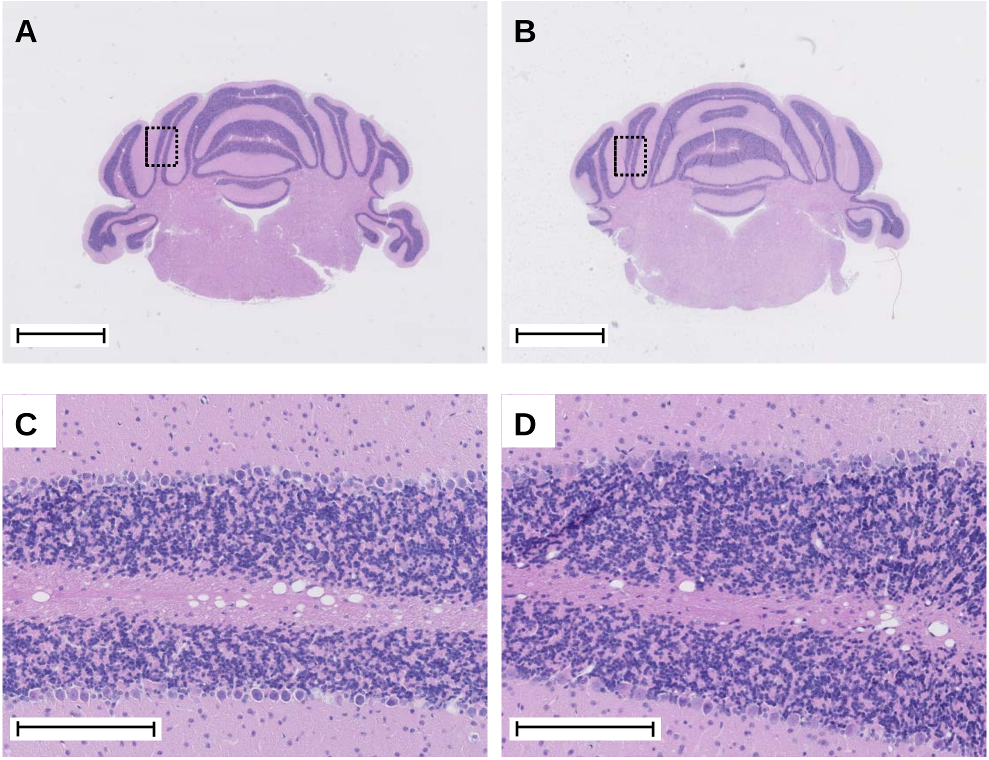

Figure S2
